# Persistence of SARS-CoV-2 Antigens in the Nasal Mucosa of Eight Patients with Inflammatory Rhinopathy for over 80 Days following Mild COVID-19 Diagnosis

**DOI:** 10.3390/v15040899

**Published:** 2023-03-31

**Authors:** Juliana Costa dos Santos, Marjory Ximenes Rabelo, Luana Mattana Sebben, Matheus Vinicius de Souza Carneiro, João Bosco Lopes Botelho, José Cardoso Neto, Anderson Nogueira Barbosa, Diego Monteiro de Carvalho, Gemilson Soares Pontes

**Affiliations:** 1 Programa de Pós-Graduação em Imunologia Básica e Aplicada, Universidade Federal do Amazonas, Avenida General Rodrigo Octávio 6200, Coroado, Manaus 69080-900, AM, Brazil; juju.costta@hotmail.com; 2 Centro Multiusuário para Análise de Fenômenos Biomédicos, Universidade do Estado do Amazonas, Avenida Carvalho Leal 1777, Cachoeirinha, Manaus 69065-000, AM, Brazil; 3 Fundação Hospital Adriano Jorge, Avenida Carvalho Leal 1778, Cachoeirinha, Manaus 69065-001, AM, Brazil; 4 Faculdade de Medicina, Universidade Federal do Amazonas, Rua Afonso Pena 1053, Manaus 69020-160, AM, Brazil; 5 Escola Superior Ciências da Saúde, Universidade do Estado do Amazonas, Avenida Carvalho Leal 1777, Cachoeirinha, Manaus 69065-000, AM, Brazil; 6 Departamento de Estatística, Universidade Federal do Amazonas, Avenida Roberto Vieira, Coroado, Manaus 69080-000, AM, Brazil; 7 Laboratório de Virologia e Imunologia, Instituto Nacional de Pesquisas da Amazônia, Avenida André Araújo 2936, Manaus 69060-001, AM, Brazil

**Keywords:** COVID-19, nasal mucosa, rhinopathy, olfactory dysfunction

## Abstract

The nasal mucosa is the main gateway for entry, replication and elimination of the SARS-CoV-2 virus, the pathogen that causes severe acute respiratory syndrome (COVID-19). The presence of the virus in the epithelium causes damage to the nasal mucosa and compromises mucociliary clearance. The aim of this study was to investigate the presence of SARS-CoV-2 viral antigens in the nasal mucociliary mucosa of patients with a history of mild COVID-19 and persistent inflammatory rhinopathy. We evaluated eight adults without previous nasal diseases and with a history of COVID-19 and persistent olfactory dysfunction for more than 80 days after diagnosis of SARS-CoV-2 infection. Samples of the nasal mucosa were collected via brushing of the middle nasal concha. The detection of viral antigens was performed using immunofluorescence through confocal microscopy. Viral antigens were detected in the nasal mucosa of all patients. Persistent anosmia was observed in four patients. Our findings suggest that persistent SARS-CoV-2 antigens in the nasal mucosa of mild COVID-19 patients may lead to inflammatory rhinopathy and prolonged or relapsing anosmia. This study sheds light on the potential mechanisms underlying persistent symptoms of COVID-19 and highlights the importance of monitoring patients with persistent anosmia and nasal-related symptoms.

## 1. Introduction

The nasal mucosa is the main port of entry for infection and reservoir for the transmissibility of respiratory viruses [1], of which SARS-CoV-2 stands out. SARS-CoV-2 belongs to the *Coronaviridae* family, genus *Betacoronavirus* [2], which causes severe acute respiratory syndrome (COVID-19) [3]. COVID-19, first reported in China in the month of December 2019 [4], quickly evolved into a pandemic and was declared by the World Health Organization (WHO) to be a public health emergency of international interest [5].

The ciliary structures of the nasal mucosa constitute the initial subcellular site for infection by SARS-CoV-2, where tropism by hair cells of the nasal epithelium occurs, a tissue rich in specific receptors that are responsible for virus-host interaction [1,6,7]. In addition, during the infection and establishment of COVID-19, this region remains rich in viral markers [8] such as glycoprotein S (spike) and nucleoprotein N, structural constituents of the viral envelope and nucleocapsid, respectively [9].

SARS-CoV-2 primarily enters into host cells through the angiotensin-converting enzyme 2 (ACE2) receptors, which are expressed on the surfaces of many different type of cells such as respiratory epithelial cells, monocytes, alveolar macrophages, neurons, endothelium and intestinal cells [10,11]. This highlights the susceptibility of multiple human tissue types to the virus [12,13]. However, the primary target of SARS-CoV-2 is the olfactory epithelium. The persistence of the virus in the tissues of recovered COVID-19 patients can lead to chronic inflammation and prolonged symptoms. For example, persistent SARS-CoV-2 RNA in the oropharyngeal tissue has been linked to an increased cellular immune response and relapsing olfactory dysfunction [14].

The cytopathic effect of SARS-CoV-2 infection on the nasal mucosa involves changes such as deciliation, short and deformed cilia [7], disorganization of ciliary structures and thinning of the ciliary layer [15]. As a result of these manifestations, there is a decrease in mucociliary clearance, accumulation of mucus and predisposition to repeated infections [16]. In addition to ciliary changes, the persistent presence of viral particles in the nasal mucosa after SARS-CoV-2 infection can promote prolonged local inflammation that may contribute to olfactory dysfunctions and cause persistent rhinitis [17].

The classical clinical manifestations of COVID-19 can range from mild to severe respiratory symptoms. Primary symptoms include fatigue, shortness of breath, coughing, loss of taste or smell, among others [18]. However, the manifestation of symptoms can be contingent upon a variety of factors, including the organ or tissue affected [19]. This can lead to multisystemic clinical manifestations and result in sequela that may persist indefinitely [20].

When symptoms persist for a period exceeding 12 weeks following recovery from SARS-CoV-2 infection, and no other underlying cause can be determined, they are classified as a post-COVID-19 condition, formerly referred to as Long-COVID-19 [14]. The persistent neurological manifestations can range from mild symptoms, such as cognitive impairment (commonly known as “brain fog”), headaches and anosmia, to severe clinical manifestations, including seizures, stroke and encephalopathy [21].

The onset of olfactory dysfunction in the context of COVID-19 is typically observed around four days after the emergence of the initial symptoms and has a close association with taste loss [22,23]. On average, the recovery period of this disorder lasts between 10–12 days. However, complete recovery may take longer than four weeks, and in some cases, it may be indefinite [14,18,21,24,25]. While it is established that prolonged symptoms are tied to SARS-CoV-2 persistence in various tissues, the precise mechanism and profile of clinical manifestations linked to viral antigen persistence in the middle nasal concha remains unclear.

The objective of this study was to examine the clinical profile and the persistence of SARS-CoV-2 viral antigens in the nasal mucociliary mucosa of patients who experienced mild COVID-19 and have ongoing inflammatory rhinopathy.

## 2. Materials and Methods

### 2.1. Ethical Approval

This study was approved by the Research Ethics Committee of the Amazonas State University (CAAE 31031720.7.0000.5016). All study participants signed the informed consent form. Confidentiality and the right to leave the study at any time were guaranteed to all participants.

### 2.2. Study Design

**Figure 1:**
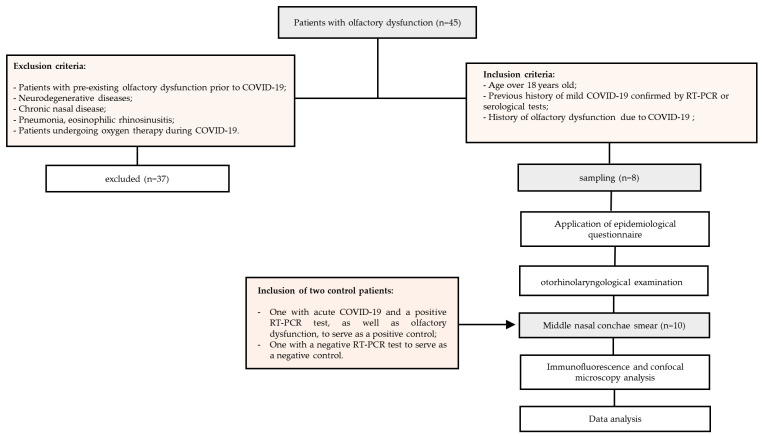
Flowchart illustrating the study’s design and the total number of individuals included.

Between June and August 2020, this study screened a total of 45 patients who visited the specialized otorhinolaryngology service at the Adriano Jorge Hospital Foundation in Manaus, Amazonas, Brazil and had reported olfactory dysfunction persisting for more than 60 days after recovering from COVID-19. Of these, eight participants (5 males and 3 females) aged between 25 and 58 years, who had developed inflammatory rhinopathy lasting more than 80 days following recovery from COVID-19, were included in the study. These individuals had previously experienced mild COVID-19, as confirmed by laboratory examinations such as RT-PCR or rapid test for SARS-CoV-2, and had reported persistent olfactory dysfunction outside of the acute phase period. Patients with pre-existing olfactory dysfunction, neurodegenerative diseases, chronic nasal disease, pneumonia, eosinophilic rhinosinusitis, or those who had undergone oxygen therapy during their COVID-19 infection were excluded from the study (Figure 1).

The included patients were submitted to a clinical-epidemiological questionnaire to obtain information regarding demographic characteristics, presence of comorbidities, use of medications, history of previous nasal surgery, previous nasal trauma, type of olfactory dysfunction, taste dysfunction, presence of nasal symptoms and time between onset of flu and olfactory symptoms and nasal sample collection (Figure 1). Patients with a positive laboratory test for COVID-19 at the time of the analyses were excluded from the study. All participants included in this study were not vaccinated against COVID-19 due to the unavailability of a vaccine during the period when the study was carried out.

### 2.3. Ear, Nose and Throat (ENT) Physical Examination

All patients underwent anterior rhinoscopy and nasal endoscopy. During the examination, the coloration of the nasal mucosa, presence or absence of hypertrophy of the nasal conchae, septal deviations and secretions were evaluated. 

The olfactory evaluation occurred via an objective olfactory test using essences. For the objective olfactory exam, the alcohol-adapted test was used [26]. A cotton pad soaked in 70% isopropyl alcohol was positioned 30 cm away from the nostril of each patient. The classification of the odor recognition threshold was defined according to the nostril distance in normosmia (30–20 cm), hyposmia (19–10 cm) and anosmia (9–0 cm). For the olfactory test with essences, the discriminative test was used [27]. During the test, 4 essences (coffee, cinnamon, citrus and mint) were presented separately, 5 cm from the nostril of each patient. The classification occurred according to the number of hits in normosmia (2–4 hits), hyposmia (1 hits) and anosmia (no recognized odor).

### 2.4. Immunofluorescence 

Nasal mucosal brushing samples from both middle conchae were obtained from all patients and SARS-CoV-2 antigen detection was done by immunofluorescence. The samples were collected with a cervical brush and fixed immediately with 4% paraformaldehyde (Sigma-Aldrich, St. Louis, MO, USA) in PBS for 24 h, permeabilized using 1% Triton X-100 (Sigma-Aldrich, St. Louis, MO, USA) in PBS and blocked with 3% BSA in PBS for 30 min. To detect the SARS-CoV-2 antigen and to stain the ciliary structure, the sample was incubated for 1 h with primary antibodies (anti-SARS-CoV-2-N—rabbit; anti-SARS-CoV-2-S—rabbit; and tubulin—mouse; Thermo Fisher Scientific, Waltham, MA, USA), followed by 4 washes with PBS. The cells were then incubated for 40 min with the secondary antibodies anti-rabbit Alexa 488 and anti-mouse Alexa 546 (Thermo Fisher Scientific, Waltham, MA, USA), followed by 4 washes with PBS. Mounting was done with the ProLong™ Gold Antifade Mountant with DAPI (Thermo Fisher Scientific, Waltham, MA, USA). The images were acquired using confocal laser scanning microscopy (Leica TCS SP8, Wetzlar, Germany), with a 63× oil immersion objective. Samples from a patient diagnosed with acute COVID-19 through RT-PCR testing served as the positive control, while the negative control comprised samples from a patient who tested RT-PCR negative for SARS-CoV-2.

### 2.5. Data Analysis

The data obtained were analyzed descriptively using the R programming language version 4.1.1 in the Integrated Development Environment, R Studio version 2021.09.0. All charts were constructed using the “ggplot2” package.

## 3. Results

The study included eight patients who had previously experienced mild COVID-19 and exhibited olfactory dysfunction. The mean length of time between the onset of the flu-like symptoms of COVID-19 and the manifestation of olfactory disorder described by the patients was 4 days (range 0 to 10 days). The mean interval between the onset of flu-like symptoms of COVID-19 and the collection of nasal samples was 83 days, ranging from 81 to 96 days. At the time of nasal sample collection, all patients included in the study were negative for SARS-CoV-2 infection.

**Figure 2:**
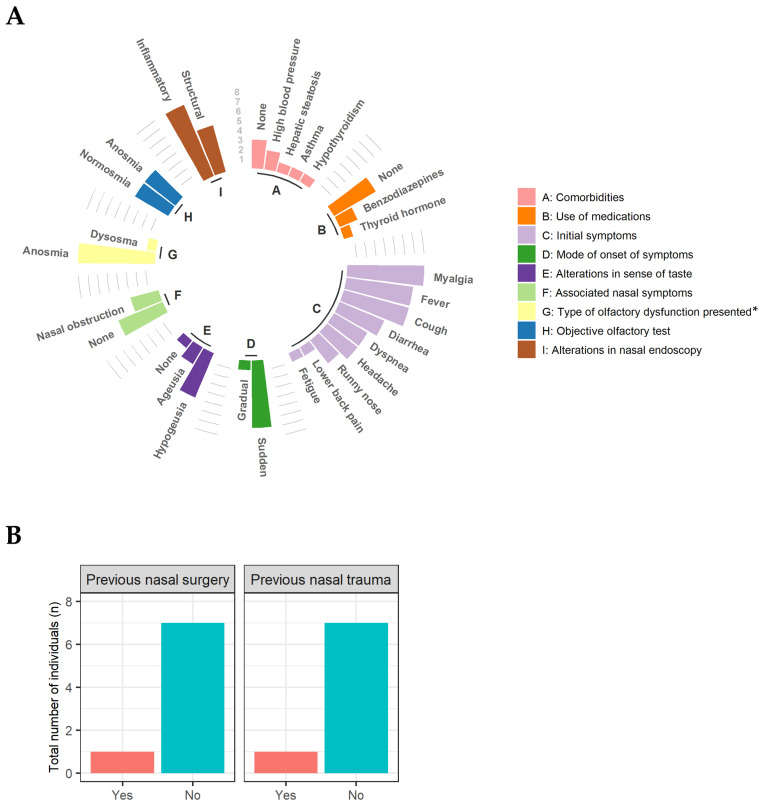
Clinical characteristics of patients complaining of mild post-COVID-19 olfactory disorders. (**A**) absolute frequency of clinical symptoms observed during application of the clinical-epidemiological questionnaire (A, B, C, D, E, F, G) and otorhinolaryngological physical examination (H, I). The letters inside the circle represent the *X*-axis and indicate which group each variable belongs to. The lines between the groups represent the *Y*-axis and indicate the absolute frequency of each variable. (**B**) Absolute frequency of surgery and previous nasal trauma. * patients’ perception of symptoms.

Most patients *(n* = 7) reported mild post-COVID-19 anosmia with sudden onset of symptoms during the acute phase of the disease, as well as changes in their sense of taste, hypogeusia (*n* = 5) and ageusia (*n* = 2) (Figure 2A). However, at the time of the study, only 50% (*n* = 4) of the patients still had the olfactory dysfunction anosmia confirmed by olfactory tests. When queried regarding additional nasal symptoms, three patients reported experiencing symptoms, particularly nasal obstruction.

Rhinoscopy and nasal endoscopy revealed that all patients in the study had clinical signs that are characteristic of inflammatory rhinopathy (edema and hypertrophy of inferior nasal conchae, hyaline rhinorrhea, pallor or mucosal hyperemia). Only one patient reported having suffered nasal trauma and having undergone nasal surgery (Figure 2B).

**Figure 3:**
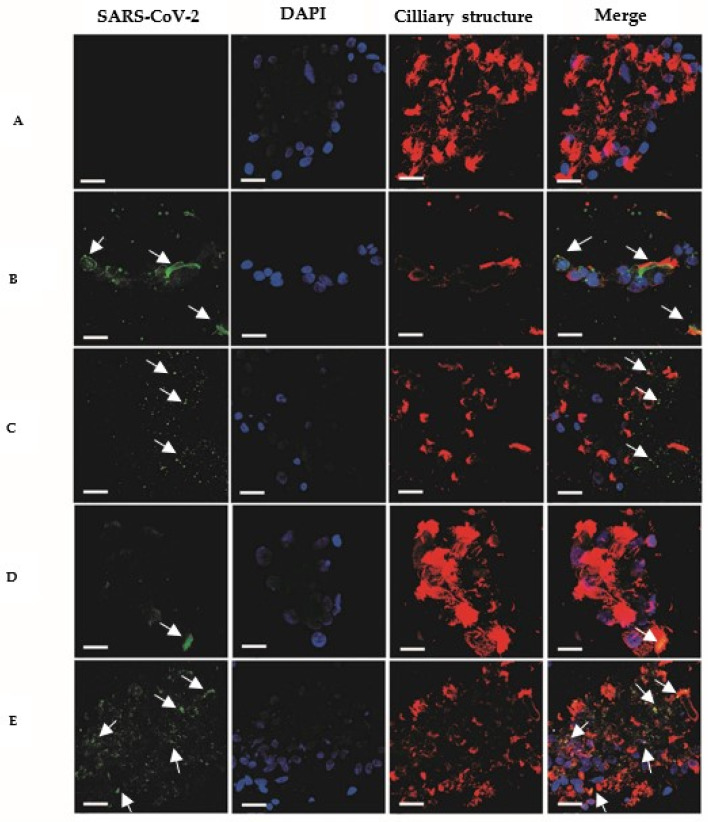
Nasal mucociliary mucosa of patients complaining of mild post-COVID-19 olfactory dysfunction. Negative control (**A**); positive control (**B**); patients (**C**–**E**). SARS-CoV-2 S/N proteins, in green; nucleic acid labeling (DAPI), in blue; ciliary structures-tubulin, in red. The “Merge” column shows all overlapping markups. Images (**C**–**E**) are representative of samples from 8 patients. The images were selected among a total of 24 images through three replicates. Scale bars represent 25 µm. Arrows indicate viral antigen (nucleocapsid and spike proteins) staining.

Immunofluorescence analysis of nasal conchae cell smears demonstrated the presence of SARS-CoV-2 glycoprotein S (spike) and nucleoprotein N in the nasal mucociliary mucosa of all study patients (Figure 3). The period during which viral antigens were detectable in the nasal mucociliary mucosa of patients ranged from 81 to 102 days after the onset of symptoms, with an average time of 90 days.

## 4. Discussion

Olfactory dysfunction is one of the most common manifestations caused by COVID-19 during mild and moderate cases [28,29]. This dysfunction usually disappears spontaneously in a period of up to 4 weeks. However, some patients may have anosmia indefinitely [24,30]. 

In this study, it was observed that the onset of olfactory dysfunction occurs on average 4 days after the onset of symptoms related to infection. In addition, almost all patients (7/8) had olfactory dysfunction associated with sense of taste. In French patients, a similar length of time for the onset of olfactory dysfunction was observed in 47% of cases, with 85% of cases presenting olfactory dysfunction associated with sense of taste (ageusia and hypogeusia) [31]. After 3 months of COVID-19, half of the patients involved in this study (*n* = 4) still had olfactory dysfunction. This chronic nasal inflammatory symptomatology observed in this study succinctly increases the risk of developing nasal and sinus disorders such as rhinitis and recurrent rhinosinusitis [32]. 

The presence of glycoprotein S (spike) and nucleoprotein (N) of SARS-CoV-2 was identified in the nasal mucociliary mucosa of all eight patients. SARS-CoV-2 presents tropism via the nasal mucosa and olfactory epithelium, where its antigens can remain for several months after diagnosis, leading to a chronic inflammatory process in patients with persistent olfactory dysfunction [33,34]. In this study, analysis by nasal endoscopy showed that all patients had chronic inflammatory rhinopathy disease, even more than 80 days after the initial diagnosis of mild COVID-19.

Olfactory dysfunction caused by SARS-CoV-2 is directly linked to several factors, including inflammation of the nasal mucosa, inflammation of the olfactory bulb, damage to the mucociliary structures and damage to the central nervous system [35]. However, the precise mechanism or pathogenesis of olfactory disorders in COVID-19 remains unclear [36,37]. A systematic review of 44 studies assessing olfactory recovery after COVID-19 showed that the recovery rate was 94.6% within one month and 85.7% after six months [25]. Our study supports the evidence that a proportion of the population experiences persistent olfactory dysfunction beyond 12 weeks after the resolution of COVID-19 [38].

The release of viral particles in the upper respiratory epithelium typically takes around 17 days [39]. However, longer periods of viral detection have been reported, with SARS-CoV-2 particles persisting in the mucosa of the upper airways for up to 83 days [40]. Our study’s findings are consistent with those of previous results, in which the average time between symptom onset and the detection of viral antigens was 90 days. Notably, we observed a maximum duration of persistence of viral antigens that exceeded the previously reported, with one patient exhibiting viral antigens in the nasal mucosa after 102 days of symptom onset.

A study investigating the interaction of SARS-CoV-2 with the olfactory system found persistent viral antigens (N protein) in the olfactory neuroepithelium of patients with olfactory dysfunction. These patients also exhibited immune and apoptotic cells and upregulation of the pro-inflammatory cytokine IL-6 similar to acute COVID-19 patients [17]. Viral antigens were also found in the nasal mucosa of a patient without olfactory dysfunction, but with less severe nasal inflammation. These results are consistent with our findings, which indicated that the persistence of viral antigens and inflammation in the middle nasal conchae could contribute to the development of persistent olfactory dysfunction in patients who have recovered from mild COVID-19.

Despite high viral loads in respiratory tissue, the SARS-CoV-2 virus has the ability to replicate in all tissues of the body, leading to persistence. For instance, in gastrointestinal tissue, viral particles have been detected up to 70 days after symptom onset [41]. Regarding the brain, the SARS-CoV-2 has the potential to persist for up to 230 days [42]. Even in cases of mild COVID-19, the virus may remain persistent in brain tissue, leading to prolonged neuroinflammatory changes and resulting in characteristic post-COVID-19 symptoms such as brain fog and sensory dysfunction [43]. In this study, all patients had mild COVID-19, yet they showed viral persistence in the middle nasal conchae mucosa, which likely resulted in prolonged inflammation and, consequently, persistent olfactory dysfunction.

The prolonged presence of viral antigens in the nasal mucosa of patients with a history of SARS-CoV-2 infection seems to be related to elevated levels of inflammatory cytokines in the olfactory mucosa [17]. Despite the observed inflammatory process, it was not possible to evaluate the serum cytokine profile of the participants in this study. Persistent nasal mucosal inflammation was also observed in 112 French patients, between 2 and 5 months post-infection with SARS-CoV-2 with and without olfactory dysfunction [44]. In the present study, the patients were followed up for a maximum of 102 days, and it was not possible to assess how long the symptoms lasted. In addition, the presence of antigens was verified transversely due to the degree of invasiveness involved in collecting the clinical sample, which did not allow us to determine how long the presence of viral antigens in the nasal mucosa lasted for.

Taken all together, these findings provide new insights into the pathophysiological mechanisms underlying persistent olfactory dysfunction in COVID-19 and may have implications for improving the clinical management and prevention of associated inflammatory and infectious complications.

## 5. Conclusions

In conclusion, this study provides evidence suggesting that persistent SARS-CoV-2 viral antigens in the mucosa of the middle nasal conchae of individuals with mild post-COVID-19 symptoms may be linked to the development of persistent inflammatory rhinopathy that may lead to prolonged or relapsing anosmia. Additionally, our findings highlight chronic olfactory dysfunction as the primary clinical manifestation observed in these cases, which has the potential to be long-lasting.

## Data Availability

Not applicable.
